# Condensation Reactions of 3-Oxo-2-arylhydrazonopropanals with Active Methylene Reagents: Formation of 2-Hydroxy- and 2-Amino-6-substituted-5-arylazonicotinates and Pyrido[3,2-c]cinnolines via 6π-Electrocyclization Reactions

**DOI:** 10.3390/molecules17066547

**Published:** 2012-05-30

**Authors:** Saleh M. Al-Mousawi, Morsy A. El-Apasery

**Affiliations:** 1 Chemistry Department, Faculty of Science, Kuwait University, P.O. Box 5969, Safat 13060, Kuwait; 2 Dyeing, Printing and Textile Auxiliaries Department, Textile Research Division, National Research Centre, Dokki, Giza 12622, Egypt

**Keywords:** arylhydrazonopropanals, arylazonicotinates, pyridazinones, cinnolines

## Abstract

3-Oxo-3-phenyl-2-(*p*-tolylhydrazono)propanal (**1a**) undergoes condensation with ethyl cyanoacetate in acetic acid in the presence of ammonium acetate to yield either 2-hydroxy-6-phenyl-5-*p*-tolylazonicotinic acid ethyl ester (**6a**) or 2-amino-6-phenyl-5-*p*-tolyl-azonicotinic acid ethyl ester (**8**), depending on the reaction conditions. Similarly, other 3-oxo-3-aryl-2-arylhydrazonopropanals **1a**,**b** condense with active methylene nitriles **2c**,**d** to yield arylazonicotinates **6b**,**c**. In contrast, 2-[(4-nitrophenyl)-hydrazono]-3-oxo-3-phenyl-propanal (**1c**) reacts with ethyl cyanoacetate to yield ethyl 6-(4-nitrophenyl)-2-oxo-2,6-dihydropyrido[3,2–c]cinnoline-3-carboxylate (**11**), via a novel 6π-electrocyclization pathway. Finally, 3-oxo-2-(phenylhydrazono)-3-*p*-tolylpropanal (**1d**) condenses with **2a–c** to yield pyridazinones **13a–c**.

## 1. Introduction

The chemistry of arylhydrazonopropanals **1** has attracted considerable attention [1,2]. These substances have proven to be valuable precursors of 3-aroylpyrzoles [3], 3-aroylpyridazine-4,6-dicaboxylic acids [4], 3-aroylcinnolines [5], as well as novel 3-aroyl-1,6-dihydropyridazines [6]. Although condensation reactions of arylhydrazonopropanals with active methylene nitriles were originally reported to afford pyridazin-6-imines [3], more recent studies in our laboratories have demonstrated that arylazonicotinates are also formed in some of these processes [7,8,9].

Because arylazonicotinates are a valuable class of arylazopyridine dyes whose chemistry has attracted some interest as new disperse dyes [10,11], it seemed of value to undertake an investigation aimed at exploring the potential utility of arylhydrazonopropanals as precursors for the preparation of these targets. A recent investigation, described below, has led to the synthesis of two different types of substances, including 2-hydroxyarylazonicotinic acid ethyl esters and 2-aminoarylazonicotinic acid ethyl esters, along with ethyl 6-(4-nitrophenyl)-2-oxo-2,6-dihydropyrido[3,2–c]cinnoline-3-carboxylate, which forms via a pathway involving a novel 6π-electrocyclization reaction.

## 2. Results and Discussion

**Scheme 1:**
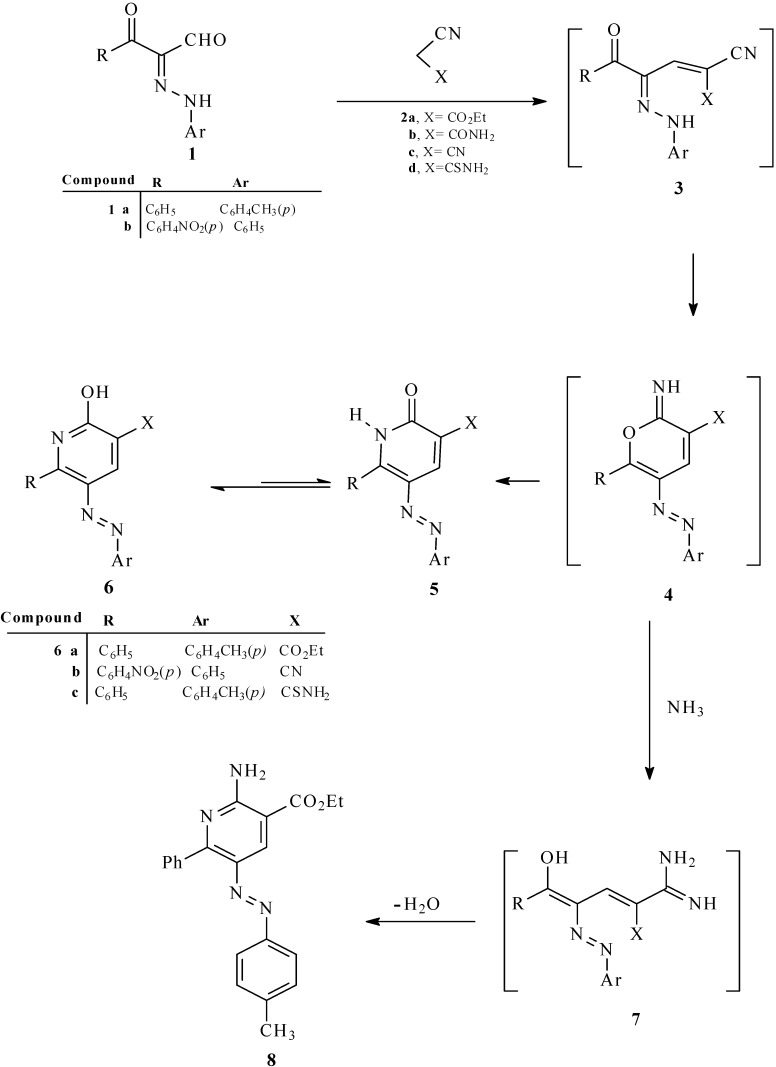
Synthesis of 2-hydroxy-6-substituted-5-arylazonicotinates derivatives **6a–c** and *2-*amino-6-phenyl-5-p-tolylazo-nicotinate **8**.

**Figure 1:**
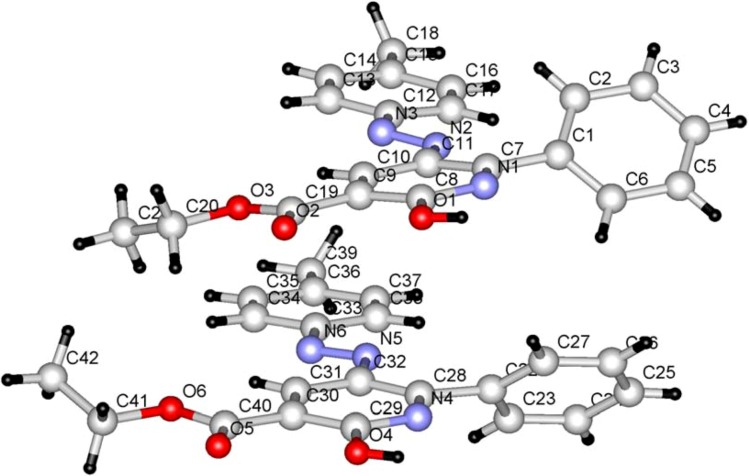
Ball and stick drawing of **6a**.

In the first phase of the current effort, we observed that reaction of the 3-oxo-3-phenyl-2-(*p*-tolylhydrazono)propanal (**1a**) with ethyl cyanoacetate in acetic acid for 30 min in the presence of a catalytic amount of ammonium acetate leads to formation of the ethyl 2-hydroxy-6-phenyl-5-*p*-tolylazonicotinate (**6a**; Scheme 1) whose structure was established by X-ray crystallographic analysis (Figure 1) [12].

**Figure 2:**
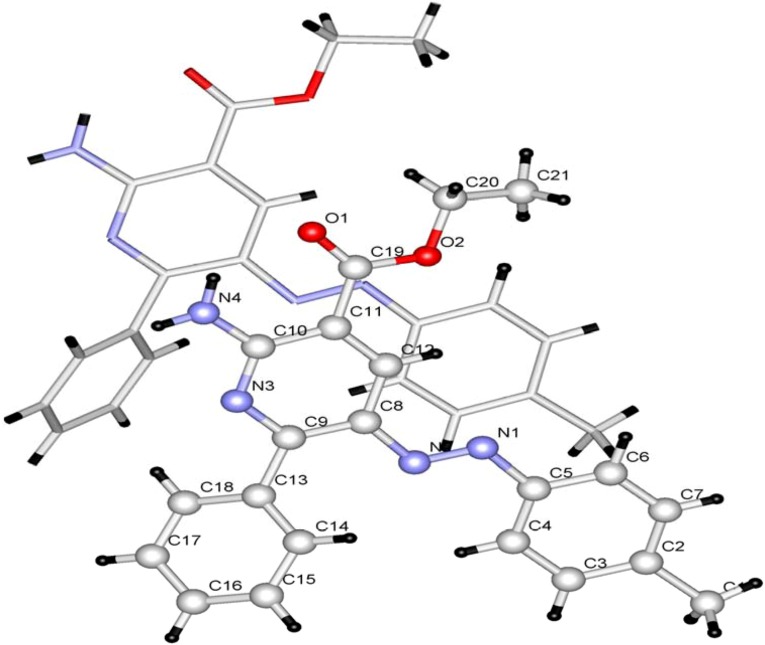
Ball and stick drawing of **8a**.

In contrast, when the condensation reaction of **1a** with ethyl cyanoacetate is conducted in the presence of excess of ammonium acetate, ethyl 2-amino-6-phenyl-5-*p*-tolylazonicotinate (**8**) is produced. The structure of **8** was also assigned by using X-ray crystallographic methods (Figure 2) [12].

It is believed that these processes involve initial reaction of **1a** with ethyl cyanoacetate to yield the hydrazono-enone **3** that then cyclizes to generate the pyran-imine **4**. In the absence of ammonium ion, **4** undergoes a Dimroth type rearrangement to yield **6a**. However, in the presence of a high concentration of ammonium acetate, pyran-imine **4** participates in a ring opening to yield amidine **7** that then cyclizes followed by water elimination to yield **8**.

In a similar manner, the 3-oxo-3-aryl-2-arylhydrazonopropanals **1a**,**b** undergo condensation reactions with other active methylene nitriles **2c**,**d **(Scheme 1) to yield the corresponding arylazonicotinates **6b**,**c** (catalytic ammonium acetate).

**Scheme 2:**
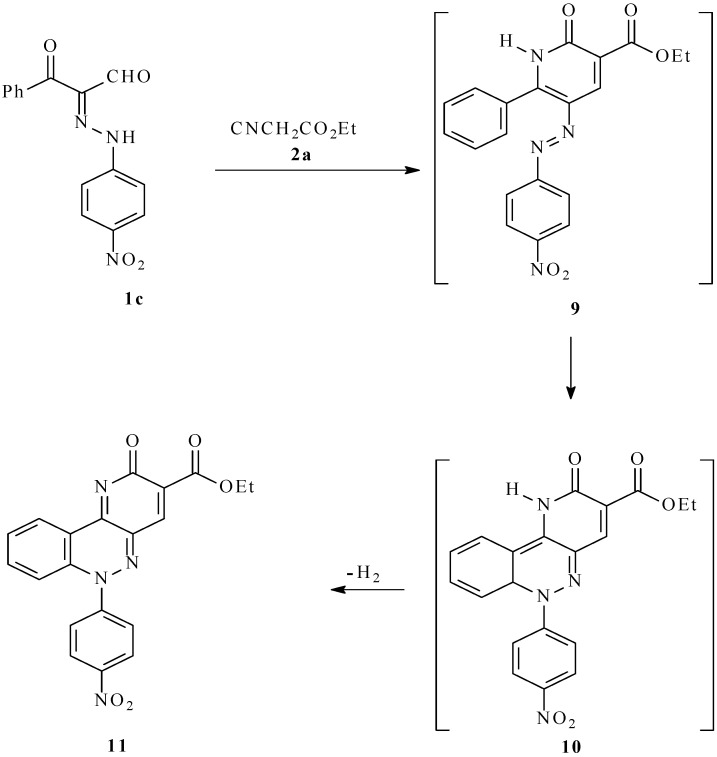
Synthesis of ethyl 6-(4-nitrophenyl)-2-oxo-2,6-dihydropyrido[3,2-c]cinnoline-3-carboxylate (**11**).

In contrast to the reactivity profiles displayed by its analogs, the 2-[(4-nitrophenyl)-hydrazono]-3-oxo-3-phenylpropanal (**1c**) reacts with ethyl cyanoacetate (90 min) to afford the novel ethyl 6-(4-nitrophenyl)-2-oxo-2,6-dihydropyrido[3,2-c]cinnoline-3-carboxylate (**11**; Scheme 2). This substance is believed to be formed via a 6π-electrocyclization reaction of the initially formed arylazonicotinate **9** (analogous to **5** in Scheme 1) that generates the tricyclic intermediate product **10**, which then aromatizes to produce the pyrido[3,2–c]cinnoline **11**. The high electrocyclization reactivity of **9** appears to be a consequence of the presence of the electron-withdrawing nitro substituent that apparently alters in a favorable way the frontier orbital interactions involved in the pericyclic process.

**Scheme 3:**
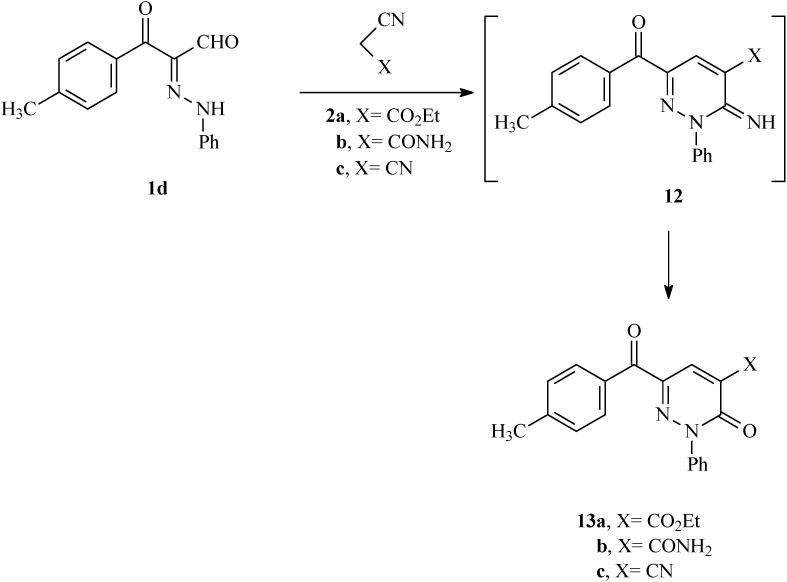
Synthesis of 6-(4-methylbenzoyl)-3-oxo-2-phenyl-2,3-dihydropyridazine derivatives **13a–c**.

**Figure 3:**
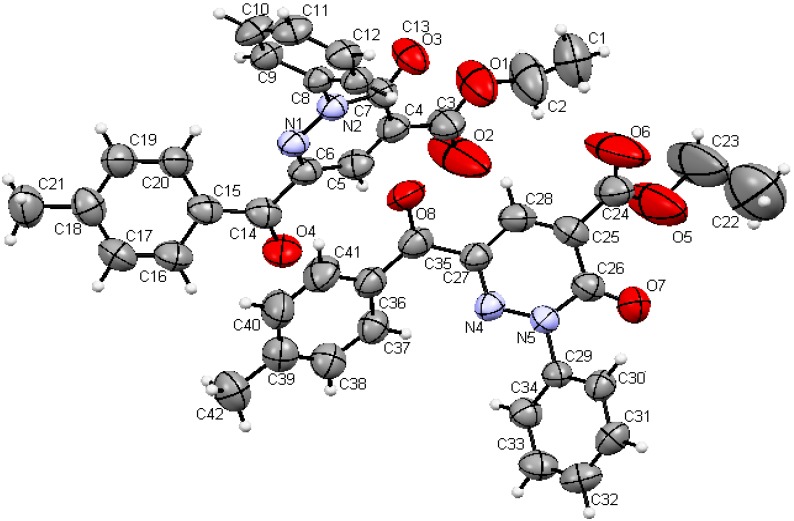
ORTEP drawing of **13a**.

In the final phase of the current effort, we observed that reactions of the 3-oxo-2-(phenylhydrazono)-3-*p*-tolylpropanal (**1d**) with active methylene nitriles **2a–c** in the presence of catalytic amounts of ammonium acetate in acetic acid for 30 min lead to the respective pyridazinones **13a–c**, which are likely formed via the intermediacy of the readily hydrolyzed imine analogs **12** (Scheme 3). The structure of **13a** was established by X-ray crystallography (Figure 3) [12].

The differences in the reactivity profiles of **1a–c** *vs.* **1d** may be a result of the decreased nucleophilicity of the aroyl carbon in the later substance, enabling cyclization of the hydrazone moiety to predominate.

Currently, we are utilizing the 2-hydroxy-and 2-aminoazonicotinates dyes as disperse dyes and applying them to polyester fabrics by using high temperature dyeing method. We are also inspecting the biological activity of these disperse dyes against Gram positive bacteria, Gram negative bacteria and yeast.

## 3. Experimental

### 3.1. General

Melting points were recorded on a Gallenkamp apparatus. IR spectra were recorded using KBr pellets on a Jasco FTIR-6300 FT-IR spectrophotometer. ^1^H- and ^13^C-NMR spectra were recorded on Bruker DPX 400 MHz or AvanceII 600 MHz super-conducting NMR spectrometers (proton spectra measured at 400, 600 MHz and carbon spectra at 100 and 150 MHz, respectively). Mass spectra were measured on a high resolution GC/MS DFS-Thermo. Microanalyses were performed on Elementar-Vario Micro cube Analyzer. X-Ray analyses were performed using a Rigaku Rapid II diffractometer. 

### 3.2. General Procedure for the Preparation of Compounds **6a–c**

Independent mixtures of **1a,b** (0.01 mol), active methylenenitrile derivatives **2a** or **2c** or **2d** (0.01 mol), and ammonium acetate (0.5 g) in acetic acid (10 mL) were stirred at reflux for 30 min (progress of the reactions was monitored by using TLC using 1:1 ethyl acetate-petroleum ether as eluent). The mixtures were cooled and then poured into ice-water. The solids that formed were collected by using filtration and crystallized from proper solvents to give **6a–c**.

*2-Hydroxy-6-phenyl-5-p-tolylazonicotinic acid ethyl ester* (**6a**). This compound was obtained as dark brown crystals (83%); mp 180–182 °C; IR (KBr): = 3401 (OH), 1692 (CO) cm^−1^; ^1^H-NMR (DMSO-d_6_): δ = 1.35 (t, 3H, *J* = 7.2 CH_3_), 2.50 (s, 3H, CH_3_), 4.36 (q, 2H, *J* = 7.2 CH_2_), 7.32 (d, 2H, *J* = 8.0 Hz arom-H), 7.48–7.50 (m, 3H, arom-H), 7.60 (d, 2H, *J* = 8.0 Hz arom-H), 7.78–7.79 (m, 2H, arom-H), 7.98 (s, 1H, OH, D_2_O exchangeable). 8.54 (s, 1H, arom-H); ^13^C-NMR (DMSO-d_6_): δ = 166.5, 161.0, 158.5, 154.5, 150.6, 142.9, 138.3, 136.5, 131.0, 130.8, 129.8, 127.4, 122.3, 105.2, 61.0, 20.9, 14.1; MS, *m/z* (%), 359 ([M-2]^+^, 100), 331 (7), 241 (10), 213 (23), 196 (12), 168 (8), 148.1 (17), 115 (14), 91 (35). HRMS: *m/z* (EI) for C_21_H_19_N_3_O_3_; calcd. 361.1417; found: 361.1417.

*2-Hydroxy-6-(4-nitrophenyl)-5-phenylazonicotinonitrile* (**6b**). This compound was obtained as a dark brown powder (91%); mp 276–278 °C; IR (KBr): = 3446 (OH), 2201 (CN) cm^−1^; ^1^H-NMR (DMSO-d_6_): 7.25–7.62 (m, 6H, arom-H, OH), δ = 7.27 (d, 1H, *J* = 8.8 Hz arom-H), 8.01–8.08 (m, 1H, arom-H), 8.20 (d, 1H, *J* = 8.8 Hz arom-H), 8.27–8.36 (m, 2H, arom-H), ^13^C-NMR (DMSO-d_6_): δ = 159.8, 152.2, 151.1, 147.6, 141.7, 139.6, 139.0, 131.1, 129.7, 129.2, 126.3, 123.4, 123.1, 114.0, MS, *m/z* (%), 343 ([M-2]^+^, 10), 313 (8), 284 (16), 207 (36), 193 (12), 150 (26), 93 (100), 77 (54). Anal. Calcd for C_18_H_11_N_5_O_3_: C, 62.61; H, 3.21; N, 20.28. Found: C, 62.78; H, 3.28; N, 20.20

*2-Hydroxy-6-phenyl-5-p-tolylazothionicotinamide* (**6c**). This compound was obtained as a dark brown powder (77%); mp 276–278 °C; IR (KBr): = 3441 (OH), 3385, 3291 (NH_2_), 1660 (CO) cm^−1^; ^1^H- NMR (DMSO-d_6_): δ = 2.26 (s, 3H, CH_3_), 7.02–8.01 (m, 9H, arom-H), 8.57 (s, 1H, OH), 8.76 (s, 1H, arom-H), 12.38 (s, 2H, NH_2_, D_2_O exchangeable). ^13^C-NMR (DMSO-d_6_): δ = 190.3, 162.2, 158.0, 152.7, 141.7, 138.8, 137.3, 135.8, 131.7, 130.6, 129.4, 129.0, 124.5, 94.0, 20.8; MS, *m/z* (%), 348 ([M]^+^, 6), 333 (7), 315 (42), 257 (4), 183 (6), 119 (100), 91 (50), 77 (20). Anal. Calcd for C_19_H_16_N_4_OS: C, 65.50; H, 4.63; N, 16.08; S, 9.20. Found: C, 65.60; H, 4.30; N, 16.43; S, 8.73.

### 3.3. 2-Amino-6-phenyl-5-p-tolylazonicotinic Acid Ethyl Ester (**8**)

Independent mixtures of **1a** (0.01 mol), ethyl cyanoacetate **2a** (0.01 mol), and ammonium acetate (3 g) in acetic acid (10 mL) were stirred at reflux for 30 min (progress of the reactions was monitored by using TLC using 1:1 ethyl acetate-petroleum ether as eluent). The mixtures were cooled and then poured into ice-water. The solids that formed were collected by using filtration and crystallized from proper solvents to give **8** as wine red crystals (83%) (mp 210–212 °C); IR (KBr): = 3402, 3275 (NH_2_) 1693 (CO) cm^−1^; ^1^H-NMR (DMSO-d_6_): δ = 1.37 (t, 3H, *J* = 7.2 CH_3_), 2.50 (s, 3H, CH_3_), 4.39 (q, 2H, *J* = 7.2 CH_2_), 7.31 (d, 2H, *J* = 8.4 Hz arom-H), 7.47–7.48 (m, 3H, arom-H), 7.98 (s, 2H, NH_2_, D_2_O exchangeable). 7.59 (d, 2H, *J* = 8.4 Hz arom-H), 7.80–7.82 (m, 2H, arom-H), 8.54 (s, 1H, arom-H);^ 13^C-NMR (DMSO-d_6_): δ = 166.2, 161.3, 159.5, 150.4, 140.6, 137.2, 136.5, 130.8, 129.8, 129.2, 127.4, 127.2, 122.3, 104.9, 61.0, 20.9, 14.1; MS, *m/z* (%), 359 ([M-1]^+^, 100), 315 (9), 290 (12), 241 (6), 213 (20), 196 (9), 168 (5), 140 (9), 105 (26), 91 (36), 77 (16). Anal. Calcd for C_21_H_20_N_4_O_2_: C, 69.98; H, 5.59; N, 15.55. Found: C, 69.99; H, 5.50; N, 15.25.

### 3.4. Synthesis of Ethyl 6-(4-nitrophenyl)-2-oxo-2,6-dihydropyrido[3,2-c]cinnoline-3-carboxylate (**11**)

A mixture of **1c** (0.01 mol), ethyl cyanoacetate (0.01 mol), and ammonium acetate (0.5 g) in acetic acid (10 mL) was stirred at reflux for 90 min (progress of the reaction was monitored by using TLC using 1:1 ethyl acetate: petroleum ether). The mixture was cooled and then poured into ice-water. The solid that formed was collected by using filtration and crystallized from dioxane to give **11**. This compound was obtained as a dark brown powder (62%); mp 148–150 °C; IR (KBr): = 1694 (CO) cm^−1^; ^1^H-NMR (DMSO-d_6_): δ = 1.35 (t, 3H, *J* = 7.2 CH_3_), 4.37 (q, 2H, *J* = 7.8 CH_2_), 7.51–7.55 (m, 3H, arom-H), 7.78–8.04 (m, 3H, arom-H), 8.28–8.38 (m, 2H, arom-H), 8.62 (s, 1H, arom-H); ^13^C-NMR (DMSO-d_6_): δ = 189.4, 166.0, 163.8, 160.2, 155.7, 147.6, 142.2, 136.9, 131.0, 130.2, 129.8, 128.2, 127.4, 125.1, 123.2, 105.3, 61.2, 14.1; MS, *m/z* (%), 390 ([M]^+^, 100), 361 (9), 321 (7), 257 (5), 241 (8), 213 (24), 196 (14), 168 (11), 140 (22), 105 (31), 77 (16). Anal. Calcd for C_20_H_14_N_4_O_5_: C, 61.54; H, 3.62; N, 14.35. Found: 61.61; H, 3.57; N, 14.58.

### 3.5. General Procedure for the Preparation of Compounds *13a–c*

Independent mixtures of **1d** (0.01 mol), active methylenenitrile derivatives **2a–c** (0.01 mol), and ammonium acetate (0.5 g) in acetic acid (10 mL) were stirred at reflux for 30 min (progress of the reactions was monitored by using TLC using 1:1 ethyl acetate-petroleum ether as eluent). The mixtures were cooled and then poured into ice-water. The solids that formed were collected by using filtration and crystallized from proper solvents to give **13a–c**.

*6-(4-Methylbenzoyl)-3-oxo-2-phenyl-2,3-dihydropyridazine-4-carboxylic acid ethyl ester* (**13a**). This compound was obtained as buff crystals (55%); mp 108–110 °C; IR (KBr): = 1746 (CO) cm^−1^; ^1^H-NMR (DMSO-d_6_): δ = 1.32 (t, 3H, *J* = 7.2 CH_3_), 2.37 (s, 3H, CH_3_), 4.34 (q, 2H, *J* = 7.2 CH_2_), 7.33 (d, 2H, *J* = 8.0 Hz arom-H), 7.45–7.49 (m, 1H, arom-H), 7.51–7.55 (m, 2H, arom-H), 7.59–7.63 (m, 2H, arom-H), 7.93 (d, 2H, *J* = 8.4 Hz arom-H), 8.31 (s, 1H, arom-H); ^13^C-NMR (DMSO-d_6_): δ = 188.3, 162.6, 155.9, 144.4, 141.6, 141.1, 132.3, 132.1, 131.0, 130.7, 129.2, 128.8, 126.0, 115.2, 61.8, 21.2, 13.9; MS, *m/z* (%), 362 ([M]^+^, 48), 315 (18), 290 (16), 261 (6), 182 (12), 119 (100), 91 (46), 77 (26). HRMS: *m/z* (EI) for C_21_H_18_N_2_O_4_; calcd. 362.1261; found: 362.1261.

*6-(4-Methylbenzoyl)-3-oxo-2-phenyl-2,3-dihydropyridazine-4-carboxylic acid amide* (**13b**). This compound was obtained as a brown powder (52%); mp 243–245 °C; IR (KBr): = 3343, 3156 (NH_2_), 1698 (CO) cm^−1^; ^1^H-NMR (DMSO-d_6_): δ = 1.37 (s, 3H, CH_3_), 7.33 (d, 2H, *J* = 8.0 Hz arom-H), 7.47–7.56 (m, 3H, arom-H), 7.63 (d, 2H, *J* = 7.8 Hz arom-H), 7.93 (d, 2H, *J* = 8.0 Hz arom-H), 8.27 (s, 2H, NH_2_, D_2_O exchangeable). 8.56 (s, 1H, arom-H); ^13^C-NMR (DMSO-d_6_): δ = 188.4, 162.1, 159.5, 144.1, 142.6, 141.1, 132.5, 132.3, 130.9, 130.7, 129.0, 128.9, 128.8, 126.1, 21.2; MS, *m/z* (%), 333 ([M]^+^, 100), 318 (15), 261 (5), 214 (5), 182 (9), 119 (100), 91 (36), 77 (20). Anal. Calcd for C_19_H_15_N_3_O_3_: C, 68.46; H, 4.54; N, 12.61. Found: 68.93; H, 4.56; N, 13.02.

*6-(4-Methylbenzoyl)-3-oxo-2-phenyl-2,3-dihydropyridazine-4-carbonitrile* (**13c**). This compound was obtained as brown powder crystals (64%); mp 186–188 °C; IR (KBr): 2209 (CN), 1656 (CO) cm^−1^; ^1^H-NMR (DMSO-d_6_): δ = 2.32 (s, 3H, CH_3_), 7.25 (d, 2H, *J* = 8.0 Hz arom-H), 7.46–7.58 (m, 5H, arom-H), 7.85 (d, 2H, *J* = 8.0 Hz arom-H), 8.60 (s, 1H, arom-H); ^13^C-NMR (DMSO-d_6_): δ = 187.6, 162.0, 156.1, 144.4, 141.6, 140.5, 138.6, 132.0, 130.7, 130.2, 126.1, 125.6, 115.2, 113.7, 21.0, MS, *m/z* (%), 315 ([M]^+^, 60), 257 (6), 211 (8), 183 (14), 119 (100), 91 (42), 77 (20). HRMS: *m/z* (EI) for C_19_H_13_N_3_O_2_; calcd. 315.1001; found: 315.1001.

## 4. Conclusions

In conclusion, in the investigation described above, we have observed that 3-oxo-2-arylhydrazonopropanals that do not possess strongly electron-withdrawing aryl substituents react with active methylene nitriles to afford 2-arylhydroxyazonicotinates and 2-arylaminoazonicotinates, in a manner that depends on the concentrations of ammonium acetate. In the case of the *p*-nitro-substituted members of this family, a facile 6π-electrocyclization reaction takes place on the hydrazono-pyridone intermediate to yield the corresponding ethyl 6-(4-nitrophenyl)-2-oxo-2,6-dihydropyrido[3,2-c]cinnoline-3-carboxylate, a likely result of a substituent effect on frontier orbital interactions that favor the pericyclic process. 
